# Impact of Obeticholic Acid Therapy on Insulin Resistance and Metabolic Parameters in Metabolic Dysfunction–Associated Steatotic Liver Disease (MASLD)

**DOI:** 10.1155/ijh/3306359

**Published:** 2026-08-06

**Authors:** Majid Almansouri, Hani Shalabi, Mohammed Karami, Omar Alharthy, Ali Kabli, Mohammad Khalil, Maan Alkhattabi, Dahlia Mirdad, Ahmed Alharthy, Ahmed Abduljabbar, Mohammed Wazzan, Hatim Alabsi, Siti S. Maidin, Humaira Waseem, Amber Hassan

**Affiliations:** ^1^ Department of Clinical Biochemistry, Faculty of Medicine, King Abdulaziz University, Jeddah, Saudi Arabia, kau.edu.sa; ^2^ Department of Internal Medicine, Faculty of Medicine, University of Jeddah, Jeddah, Saudi Arabia, uj.edu.sa; ^3^ Department of Clinical Physiology, Faculty of Medicine, King Abdulaziz University, Jeddah, Saudi Arabia, kau.edu.sa; ^4^ Department of Internal Medicine, Faculty of Medicine, University of Taibah, Madina, Saudi Arabia, taibahu.edu.sa; ^5^ Department of Physiology, Faculty of Medicine, King Abdulaziz University, Rabigh, Saudi Arabia, kau.edu.sa; ^6^ Department of Radiology, Faculty of Medicine, King Abdulaziz University, Jeddah, Saudi Arabia, kau.edu.sa; ^7^ Department of Internal Medicine, Faculty of Medicine, King Abdulaziz University, Rabigh, Saudi Arabia, kau.edu.sa; ^8^ Department of Basic Medical Sciences, Faculty of Medicine, University of Jeddah, Jeddah, Saudi Arabia, uj.edu.sa; ^9^ Department of Data Science, INTI International University, Nilai, Malaysia, newinti.edu.my; ^10^ Department of Quality Enhancement Cell, Fatima Jinnah Medical University, Lahore, Pakistan, fjmc.edu.pk; ^11^ Department of Neurogenetics, Istituto Neurologico Nazionale a Carattere Scientifico, IRCCS, Pavia, Italy, ircc.it

**Keywords:** insulin resistance, metabolic dysfunction–associated steatotic liver disease, obeticholic acid

## Abstract

**Background:**

Metabolic dysfunction–associated steatotic liver disease (MASLD) is a common metabolic liver condition primarily managed through lifestyle modification. Obeticholic acid (OCA), a farnesoid X receptor agonist, has shown therapeutic benefits in steatohepatitis, yet its short‐term metabolic effects remain unclear. This study compared OCA plus lifestyle modification versus lifestyle intervention alone in adults with MASLD.

**Methods:**

This quasi‐experimental study included 360 adults aged 18–60 years with ultrasound‐diagnosed MASLD and insulin resistance (HOMA − IR > 2.0). Patients with diabetes mellitus, viral hepatitis, cirrhosis, advanced fibrosis, ischemic heart disease or LDL − C > 160 mg/dL were excluded. Participants received either OCA (10 mg/day) plus standardised lifestyle modification or lifestyle intervention alone for 3 months. Anthropometric, biochemical and hepatic parameters were evaluated at baseline and follow‐up, with ANCOVA performed to adjust for baseline covariates.

**Results:**

Baseline demographic and metabolic characteristics were comparable between groups. After 3 months, the OCA plus lifestyle group demonstrated significantly greater improvements in ALT (36.65 ± 20.57 vs. 48.19 ± 21.35 U/L; *p* < 0.001), fatty liver index (55.99 ± 12.60 vs. 63.08 ± 11.89; *p* < 0.001) and HEPAMET score (0.33 ± 0.13 vs. 0.41 ± 0.13; *p* < 0.001), along with a greater reduction in body weight (77.28 ± 12.07 kg vs. 80.19 ± 12.37 kg; *p* = 0.024). Postintervention HOMA‐IR was significantly lower with OCA plus lifestyle than lifestyle alone (2.46 ± 0.92 vs. 2.96 ± 1.06; mean difference 0.50; *p* < 0.001). LDL‐C increased significantly in the OCA group (139.50 ± 15.74 mg/dL vs. 131.96 ± 16.36 mg/dL; *p* < 0.001).

**Conclusion:**

Over 3 months, OCA combined with lifestyle modification improved liver enzymes, steatosis and fibrosis‐related surrogate indices, and modestly reduced insulin resistance. Careful lipid monitoring is recommended, and longer term studies are required to clarify the overall metabolic and cardiovascular implications.

## 1. Introduction

Metabolic dysfunction–associated steatotic liver disease (MASLD) is currently recognised as the most prevalent chronic liver disease worldwide. It represents a major global health challenge because of its strong association with obesity, insulin resistance, Type 2 diabetes mellitus (T2DM), dyslipidaemia, hypertension and other cardiometabolic disorders [1–3]. The recently adopted nomenclature replaced the previous term nonalcoholic fatty liver disease (NAFLD) with MASLD to better reflect the central role of metabolic dysfunction in disease pathogenesis and progression. MASLD is defined by the presence of hepatic steatosis in individuals with at least one cardiometabolic risk factor and without alternative causes of steatotic liver disease, including significant alcohol consumption or secondary hepatic disorders [4–6]. Histologically, MASLD encompasses a spectrum ranging from isolated steatosis to metabolic dysfunction–associated steatohepatitis (MASH), the progressive form characterised by hepatocellular ballooning, lobular inflammation and varying degrees of fibrosis [7–9]. Importantly, fibrosis stage remains the strongest predictor of liver‐related morbidity, cirrhosis, hepatocellular carcinoma and overall mortality in patients with MASLD [1, 6].

The global prevalence of MASLD has risen substantially over the past decade and currently affects nearly one‐third of the adult population worldwide [1]. The disease is closely linked to metabolic syndrome and may both contribute to and result from insulin resistance and cardiometabolic dysfunction [10]. Patients with MASLD are at increased risk of cardiovascular disease, chronic kidney disease and extrahepatic malignancies, whereas cardiovascular disease remains the leading cause of death in this population [1, 3]. The progression from simple steatosis to MASH and advanced fibrosis is driven by multiple interconnected mechanisms, including lipotoxicity, oxidative stress, inflammatory signalling, mitochondrial dysfunction and alterations in the gut–liver axis, supporting the current ‘multiple‐hit’ hypothesis of disease pathogenesis [8, 9]. Dietary factors, particularly excessive fructose and sugar intake, also play a major role in hepatic fat accumulation and metabolic dysregulation [11].

Current diagnostic strategies for MASLD increasingly emphasise noninvasive evaluation and early identification of patients at risk for advanced fibrosis. Abdominal ultrasonography remains the first‐line imaging modality for detecting hepatic steatosis because of its accessibility and cost‐effectiveness [5, 6]. However, because liver enzymes may remain normal despite advanced disease, modern clinical pathways now incorporate noninvasive fibrosis assessment tools, including the Fibrosis‐4 index (FIB‐4), nonalcoholic fatty liver disease fibrosis score (NFS), enhanced liver fibrosis (ELF) testing, vibration‐controlled transient elastography (VCTE) and magnetic resonance‐based techniques for fibrosis stratification and disease monitoring [6, 12]. Liver biopsy remains the gold standard for definitive diagnosis and staging of MASH, although its invasive nature limits routine clinical use [2, 7].

Current guidelines recommend case‐finding strategies among high‐risk individuals, particularly those with obesity, T2DM, metabolic syndrome or persistently elevated liver enzymes [1–4]. Lifestyle modification remains the cornerstone of MASLD management. Weight reduction through dietary intervention and regular physical activity has consistently improved hepatic steatosis, insulin resistance and liver‐related outcomes [2, 12]. Mediterranean dietary patterns, antioxidant supplementation, probiotics, omega‐3 fatty acids and nutraceutical interventions have also shown beneficial metabolic and hepatic effects in patients with MASLD [2, 7, 12]. Nevertheless, pharmacological therapy for MASH has evolved substantially in recent years, with increasing interest in therapies targeting metabolic, inflammatory and fibrotic pathways [13]. Among these therapeutic targets, the farnesoid X receptor (FXR) has attracted considerable attention for its central role in regulating bile acid homeostasis, lipid metabolism, glucose homeostasis and inflammatory signalling [14]. Obeticholic acid (OCA), a potent FXR agonist, demonstrated histological improvement in patients with MASH in the FLINT trial, supporting the therapeutic potential of FXR‐directed therapy [15]. FXR activation modulates hepatic lipid metabolism by suppressing sterol regulatory element‐binding protein 1c (SREBP1c), apolipoprotein expression and triglyceride‐rich lipoprotein production, thereby reducing hepatic lipogenesis and very low‐density lipoprotein secretion [16, 17]. However, OCA therapy has also been associated with increases in low‐density lipoprotein cholesterol (LDL‐C) and reductions in high‐density lipoprotein cholesterol (HDL‐C), raising concerns regarding cardiovascular safety in a population already predisposed to atherosclerotic disease [18, 19]. FXR‐mediated alterations in lipoprotein metabolism may influence apolipoprotein production, LDL heterogeneity and cholesteryl ester transfer protein activity, potentially contributing to an atherogenic lipid profile. Traditional lipid profiles may not fully capture the atherogenic burden in patients with MASLD. Advanced lipoprotein profiling and assessment of lipoprotein particle subfractions have demonstrated improved predictive performance for cardiovascular events compared with conventional lipid measurements, particularly in individuals with insulin resistance and metabolic syndrome [20, 21].

This study is aimed at evaluating the short‐term effects of OCA (10 mg/day) combined with standardised lifestyle modification on insulin resistance, hepatic indices and metabolic parameters in adults with ultrasound‐diagnosed MASLD compared with lifestyle modification alone, while also examining the metabolic and lipid‐related implications of FXR‐directed therapy in this high‐risk cardiometabolic population.

## 2. Methodology

### 2.1. Study Design and Eligibility Screening

This quasi‐experimental study was conducted at Sir Ganga Ram Hospital from August 2024 to August 2025. Eligible participants were consecutively recruited from the outpatient hepatology/internal medicine clinics. Ethical approval was obtained from the Institutional Review Board (Approval No. 210‐Synopsis/Pediatric‐III/FJ/ERC) prior to participant recruitment, and written informed consent was obtained from all participants prior to enrollment.

Participants were sequentially allocated to treatment groups based on physician‐directed clinical decisions and patient preferences, consistent with the study′s quasi‐experimental design. Although this approach may introduce selection bias, baseline demographic and metabolic characteristics were comparable between groups.

Individuals with liver cirrhosis, Grade 3 fatty liver on ultrasound, advanced fibrosis based on the HEPAMET score, ischemic heart disease, strong family history of premature cardiac death, LDL cholesterol > 160 mg/dL, positive viral hepatitis or those unwilling to follow lifestyle modification were excluded. Grade 3 steatosis was excluded to minimise the inclusion of patients with potentially advanced liver disease requiring more intensive specialised management.

Information regarding concomitant medications, including statins, antihypertensive agents, metformin, glucagon‐like peptide‐1 (GLP‐1) receptor agonists, sodium‐glucose cotransporter‐2 (SGLT2) inhibitors, pioglitazone, vitamin E supplementation and weight‐loss medications, was not systematically collected as part of the study protocol. Consequently, the potential influence of these therapies on metabolic outcomes could not be evaluated and is acknowledged as a study limitation.

Eligible patients were invited to undergo early‐morning blood sampling to assess fasting plasma glucose and fasting serum insulin levels. The homeostatic model assessment of insulin resistance (HOMA‐IR) was used to calculate insulin resistance. The study′s objectives, procedures, potential risks and benefits were explained to patients with HOMA − IR > 2.0, and written informed consent was obtained before enrollment. The sample size was calculated based on the between‐group difference in change in insulin resistance, as measured by HOMA‐IR.

MASLD diagnosis was established using abdominal ultrasonography together with accepted metabolic diagnostic criteria. Ultrasonography was selected because it represents the standard first‐line imaging modality in routine clinical practice. However, advanced imaging techniques including VCTE (FibroScan), MRI‐proton density fat fraction (MRI‐PDFF), magnetic resonance elastography (MRE) and liver biopsy were not incorporated into the study protocol. Ultrasonographic examinations were performed by experienced consultant radiologists using standardised diagnostic criteria including increased hepatic echogenicity relative to the renal cortex, posterior beam attenuation and reduced visualisation of intrahepatic vessels. Because of the pragmatic clinical design, radiologists were not blinded to treatment allocation. Interobserver variability was not formally assessed.

### 2.2. Sample Size Calculation

The sample size was calculated based on the expected between‐group difference in change in insulin resistance as assessed by HOMA‐IR. Based on previously published randomised controlled trials of OCA, a mean difference of 13 units with a pooled standard deviation of approximately 41 was assumed, corresponding to a small‐to‐moderate effect size (Cohen^′^s *d* = 0.32) [22]. To detect this difference with 80% power at a two‐sided significance level of 0.05, a minimum of 155 participants per group was required. Allowing for an anticipated attrition rate of 15%, the total planned sample size was 360 participants.

### 2.3. Intervention

Participants in Group A were treated with OCA 10 mg/day for 3 months, plus lifestyle modification advice, and Group B were treated with lifestyle modification advice for 3 months only. Lifestyle modification consisted of standardised counselling delivered by a qualified nutritionist. Participants received individualised dietary advice targeting an approximate daily energy deficit of 500–750 kcal, emphasising a Mediterranean‐style dietary pattern rich in vegetables, fruits, whole grains, lean proteins and unsaturated fats while limiting refined carbohydrates and saturated fats. Participants were encouraged to perform at least 150 min/week of moderate‐intensity aerobic exercise together with resistance exercise twice weekly. Lifestyle counselling was reinforced during every follow‐up visit, and adherence was evaluated through attendance at scheduled visits and participant self‐report.

The data collected during the baseline phase involved demographic factors (age and gender) and anthropometric measurements, including body weight, body mass index and waist circumference. Fasting plasma glucose, fasting serum insulin, HOMA‐IR, fasting lipid profile, alanine aminotransferase (ALT), serum uric acid, fatty liver index (FLI) and HEPAMET were biochemical parameters that were measured at baseline.

### 2.4. Outcome Measures

During the 3‐month intervention, a follow‐up assessment was conducted. The reassessment of body weight, body mass index, waist circumference, fasting lipid profile and serum uric acid was performed monthly. Liver function tests were performed every 2 weeks during the first month and every 1 month thereafter. To measure the effects of treatment, fasting plasma glucose, fasting serum insulin levels, HOMA‐IR, FLI and HEPAMET score were recalculated at the conclusion of the 3‐month treatment. Adverse events were monitored among participants throughout the research period. Every patient was informed about the possible side effects of OCA, such as pruritus, abdominal pain and lipid profile changes. In the presence of pruritus, the condition was mild and treated with oral cetirizine 10 mg daily. Treatment and lifestyle changes were reinforced at every follow‐up visit.

### 2.5. Statistical Analysis

Continuous variables were expressed as mean ± SD. Between‐group comparisons were performed using independent *t*‐tests. ANCOVA was used to evaluate adjusted group differences for 3‐month HOMA‐IR, adjusting for baseline HOMA‐IR, age and BMI. A *p* value < 0.05 was considered statistically significant.

## 3. Results

At baseline, the distribution of demographic and anthropometric characteristics was comparable between the two study groups. The lifestyle modification group comprised 98 males (54.4%) and 82 females (45.6%). In comparison, the OCA plus lifestyle group comprised 82 males (45.6%) and 98 females (54.4%), resulting in an equal overall gender distribution (50% males and 50% females). The mean age was similar between the lifestyle group (39.71 ± 12.41 years) and the OCA plus lifestyle group (39.30 ± 12.54 years). Anthropometric measurements were also comparable, with a mean body weight of 82.11 ± 12.33 kg in the lifestyle group and 81.36 ± 12.26 kg in the intervention group, a mean body mass index of 29.30 ± 3.91 kg/m^2^ and 29.51 ± 3.79 kg/m^2^, respectively, and a mean waist circumference of 102.41 ± 9.75 cm and 103.06 ± 10.49 cm. These findings indicate that both groups were well matched at baseline with respect to gender distribution and key anthropometric variables (Table 1).

**Table 1:** Baseline demographic and clinical characteristics of the study participants.

Characteristic	Lifestyle group (*n* = 180)	*O* *C* *A* + *l* *i* *f* *e* *s* *t* *y* *l* *e* *g* *r* *o* *u* *p* (*n* = 180)
**Gender,** **n** **(%)**		
**Male**	98 (54.4)	82 (45.6)
**Female**	82 (45.6)	98 (54.4)
**Age (years),** **m** **e** **a** **n** ± **S** **D**	39.71 ± 12.41	39.30 ± 12.54
**Body weight (kg),** **m** **e** **a** **n** ± **S** **D**	82.11 ± 12.33	81.36 ± 12.26
**BMI (kg/m** ^**2**^ **),** **m** **e** **a** **n** ± **S** **D**	29.30 ± 3.91	29.51 ± 3.79
**Waist circumference (cm),** **m** **e** **a** **n** ± **S** **D**	102.41 ± 9.75	103.06 ± 10.49

**Figure 1:**
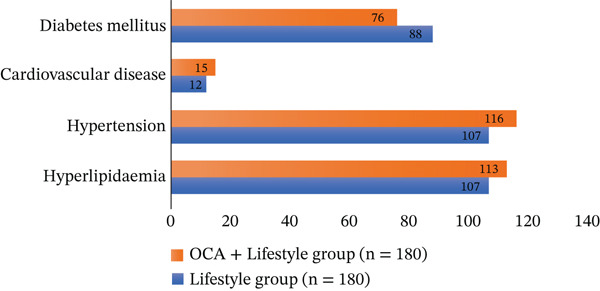
Baseline comorbidities of the study groups.

At baseline, hyperlipidaemia was present in 107 patients (59.4%) in the lifestyle‐only group and 113 patients (62.8%) in the OCA plus lifestyle group. Hypertension was observed in 107 participants (59.4%) in the lifestyle group and 116 (64.4%) in the intervention group. Cardiovascular disease was relatively uncommon, affecting 12 patients (6.7%) in the lifestyle group and 15 patients (8.3%) in the OCA plus lifestyle group. At baseline, the prevalence of key cardiometabolic comorbidities was comparable between groups. Hyperlipidaemia was present in 107 participants (59.4%) in the lifestyle‐only group and 113 (62.8%) in the OCA plus lifestyle group. Hypertension was present in 107 (59.4%) versus 116 (64.4%), respectively. Cardiovascular disease was uncommon (12 [6.7%] vs. 15 [8.3%]). Overall, baseline comorbidity profiles were balanced prior to intervention (Figure 1).

Baseline metabolic and insulin‐resistance parameters were similar between the lifestyle‐only group and the OCA plus lifestyle group. Equal levels of fasting glucose, insulin, HOMA‐IR, lipid profile, liver enzymes and noninvasive fibrosis‐related surrogate indices indicate that the two groups had similar metabolic profiles before therapy commenced; thus, the postintervention results could be compared appropriately (Table 2).

**Table 2:** Baseline insulin resistance and metabolic parameters.

Parameter	Lifestyle group (*n* = 180)	*O* *C* *A* + *l* *i* *f* *e* *s* *t* *y* *l* *e* *g* *r* *o* *u* *p* (*n* = 180)
**Fasting glucose (mmol/L)**	5.71 ± 0.64	5.80 ± 0.68
**Fasting insulin (pmol/L)**	80.19 ± 30.76	84.57 ± 31.36
**HOMA-IR**	3.40 ± 1.37	3.61 ± 1.34
**LDL cholesterol (mg/dL)**	134.20 ± 15.16	134.89 ± 13.72
**ALT (U/L)**	59.46 ± 17.86	59.85 ± 17.80
**Fatty liver index**	71.68 ± 11.32	70.50 ± 10.91
**HEPAMET score**	0.456 ± 0.125	0.458 ± 0.122

*Note:* HOMA‐IR was calculated using fasting insulin converted from pmol/L to *μU*/mL (*μ* *U*/mL = pmol/L ÷ 6).

Patients who were treated with OCA in combination with lifestyle modification achieved significantly better improvements in several metabolic and hepatic parameters after 3 months of intervention than the patients who received lifestyle modification alone. There were significant decreases in body weight and substantial changes in ALT levels, HEPAMET score and FLI in the intervention group, indicating improvements in liver enzymes and favourable changes in validated noninvasive fibrosis‐related surrogate indices. The study showed no statistically significant differences in BMI or waist circumference. The HOMA‐IR between the two groups showed an overall trend favouring the OCA group. It is important to note that LDL cholesterol levels were elevated in the OCA group, as expected given the drug′s lipid‐modifying properties. These results indicate that OCA therapy, when combined with lifestyle modification, produces significant changes in liver‐related metabolic parameters. However, the effect on insulin resistance is relatively small in the short‐term follow‐up period (Table 3).

**Table 3:** Comparison of metabolic and insulin resistance parameters between groups after 3 months of treatment.

Parameter	Lifestyle group (*n* = 180)	*O* *C* *A* + *l* *i* *f* *e* *s* *t* *y* *l* *e* *g* *r* *o* *u* *p*(*n* = 180)	Mean difference (95% CI)	*p* value
Body weight (kg)	80.19 ± 12.37	77.28 ± 12.07	2.91 (0.38–5.45)	0.024
BMI (kg/m^2^)	28.58 ± 3.89	27.96 ± 3.88	0.62 (−0.19 to 1.43)	NS
Waist circumference (cm)	99.85 ± 9.74	98.21 ± 10.71	1.64 (−0.48 to 3.76)	NS
HOMA‐IR	2.96 ± 1.06	2.46 ± 0.92	0.50 (0.30–0.71)	< 0.001
LDL cholesterol (mg/dL)	131.96 ± 16.36	139.50 ± 15.74	−7.54 (−10.87 to −4.21)	< 0.001
ALT (U/L)	48.19 ± 21.35	36.65 ± 20.57	11.54 (7.19–15.89)	< 0.001
HEPAMET score	0.41 ± 0.13	0.33 ± 0.13	0.08 (0.05–0.10)	< 0.001
Fatty liver index	63.08 ± 11.89	55.99 ± 12.60	7.09 (4.55–9.63)	< 0.001

OCA was generally well tolerated during the 3‐month intervention. Mild pruritus was the most commonly reported adverse event and was successfully managed with oral cetirizine without treatment interruption. No treatment discontinuations, serious adverse events, hepatic decompensation, gallstone‐related complications, clinically significant laboratory abnormalities or treatment withdrawals were observed during the follow‐up period. However, because comprehensive adverse‐event reporting was not a predefined study outcome, detailed safety analyses were not performed.

After adjustment for baseline HOMA‐IR, age and body mass index, a statistically significant difference in HOMA‐IR at 3 months was observed between the two study groups. The OCA plus lifestyle group exhibited significantly lower adjusted HOMA‐IR as compared with the lifestyle‐only group (adjusted mean difference *β* = 0.94; 95% CI: 0.79–1.08, *p* = 0.001), achieved with a moderate effect size (partial *η* ^2^ = 0.311). Baseline HOMA‐IR was a significant independent predictor of posttreatment HOMA‐IR, and age and baseline BMI made no significant contributions (Table 4).

**Table 4:** ANCOVA for HOMA‐IR at 3 months adjusted for baseline HOMA‐IR, age and BMI.

Effect	*β* (adjusted)	95% CI for *β*	*F*	*p* value	Partial *η* ^2^
**Group (lifestyle vs.** **O** **C** **A** + **l** **i** **f** **e** **s** **t** **y** **l** **e** **)**	0.94	0.79 to 1.08	160.27	< 0.001	0.311
**Baseline HOMA-IR**	0.997	0.988–1.006	47 965.93	< 0.001	0.993
**Age (years)**	0.002	−0.004 to 0.008	0.49	0.482	0.001
**Baseline BMI (kg/m** ^**2**^ **)**	0.018	−0.001 to 0.037	3.55	0.060	0.010

*Note:* Model *R* ^2^ = 0.993 (adjusted *R* ^2^ = 0.993), the adjusted regression coefficient is denoted by 85.45. HOMA‐IR after 3 months is the dependent variable, treatment group is the fixed factor and baseline HOMA‐IR, age and baseline BMI are covariates. Partial eta squared (*η* ^2^) represents effect size. The baseline estimates of adjusted means were HOMA − IR = 21.04, age = 39.5 years and BMI = 29.4 kg/m^2^.

**Figure 2:**
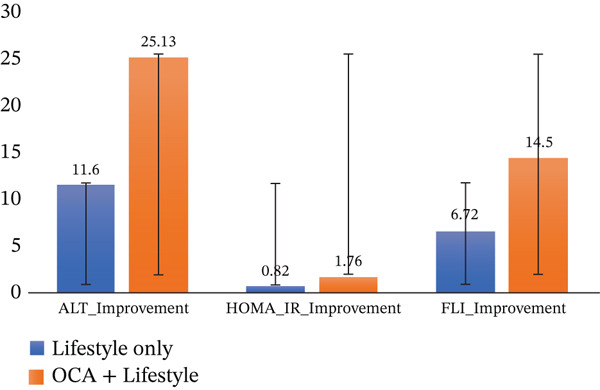
Mean improvement from baseline in metabolic and insulin resistance parameters after 3 months of intervention.

The bar chart illustrates the mean improvement from baseline in ALT. The bar chart represents the mean improvement of baseline results of ALT, insulin resistance (HOMA‐IR) and FLI in the lifestyle‐only group and the OCA plus lifestyle group after the 3‐month intervention. Patients treated with OCA, in addition to lifestyle modification, showed a greater reduction in ALT levels than those treated with lifestyle modification alone, indicating a stronger reduction in hepatic inflammation. The OCA group also showed a significantly greater improvement in the FLI, indicating greater amelioration of steatosis‐associated metabolic risk. Patients receiving OCA plus lifestyle modification showed a greater reduction in ALT compared with lifestyle modification alone, suggesting a greater improvement in liver inflammation over the 3‐month intervention (Figure 2).

## 4. Discussion

The present quasi‐experimental study demonstrates that OCA, when combined with lifestyle modification, produces significant short‐term improvements in liver enzymes and validated noninvasive fibrosis‐related surrogate indices in patients with MASLD. However, these hepatic benefits were accompanied by a statistically significant increase in LDL‐C. Although LDL‐C increased significantly, the present study did not collect data regarding statin initiation or the proportion of patients exceeding clinically relevant LDL thresholds after treatment. The clinical interpretation of these lipid alterations requires careful consideration because cardiovascular disease remains the leading cause of mortality among patients with MASLD, and extensive genetic, epidemiological and interventional evidence has established LDL particles as direct causal mediators of atherosclerotic cardiovascular disease (ASCVD) [1, 3, 18]. Consequently, any pharmacological intervention that increases circulating LDL‐C should be interpreted within the broader context of cardiometabolic risk stratification and long‐term cardiovascular outcomes.

The present findings are broadly consistent with previous clinical investigations evaluating OCA in MASLD/MASH. The FLINT trial demonstrated improvements in liver histology and serum aminotransferase concentrations following OCA treatment, although increases in LDL cholesterol and pruritus were also frequently observed [23]. Similarly, the REGENERATE trial reported improvements in fibrosis among selected patients while confirming the need for careful monitoring of treatment‐related dyslipidaemia and adverse events [24]. Although our study relied on noninvasive surrogate indices rather than histological assessment, the observed improvements in liver‐related parameters, together with increased LDL‐C, are consistent with findings from these landmark clinical trials.

In the present study, LDL‐C increased significantly in the OCA‐treated group compared with lifestyle modification alone after 3 months of therapy. The 3‐month follow‐up period was sufficient to evaluate early biochemical and metabolic changes but insufficient to assess long‐term fibrosis progression or regression, the durability of improvements in insulin resistance, cardiovascular outcomes or long‐term safety associated with OCA. Previous mechanistic studies have demonstrated that FXR activation influences lipoprotein metabolism through altered bile acid synthesis and remodelling of lipoproteins, providing a biologically plausible explanation for the increase in LDL‐C observed in our cohort [16–21].

Additionally, FXR activation suppresses bile acid synthesis from cholesterol, reducing cholesterol catabolism and potentially decreasing hepatic LDL receptor–mediated clearance. Experimental studies involving natural FXR agonists demonstrated reduced plasma LDL clearance under these metabolic conditions [23]. Therefore, the combination of increased LDL particle generation and impaired LDL clearance provides a biologically plausible explanation for the sustained LDL elevation observed in the current cohort. Similar lipid‐related findings have also been reported in trials involving FXR‐related pathways and fibroblast growth factor 19 (FGF19)–based therapies in MASH populations, where concomitant statin therapy partially mitigated treatment‐induced dyslipidaemia [18, 19]. These findings highlight the importance of lipid monitoring and consideration of adjunctive lipid‐lowering therapy during FXR agonist treatment.

The present study also demonstrated modest but statistically significant improvement in insulin resistance after adjustment for baseline covariates. Nevertheless, the magnitude of HOMA‐IR reduction remained limited, suggesting that the early hepatic improvements associated with OCA therapy may occur partially independently of major systemic insulin sensitisation. Current understanding of MASLD pathophysiology indicates that FXR activation exerts direct hepatic metabolic effects involving suppression of hepatic lipogenesis, modulation of inflammatory signalling and improvement of bile acid homeostasis [8, 14]. Therefore, the observed reductions in ALT, FLI and HEPAMET score in the present study may primarily reflect direct hepatic metabolic modulation rather than profound systemic metabolic improvement.

The link between VLDL metabolism and LDL subclass formation helps explain lipid changes in this cohort. Apolipoprotein B‐100 kinetics influence VLDL production and LDL distribution. Although advanced lipoprotein subclass analysis was not performed, previous studies suggest that FXR activation may alter LDL particle composition. Further studies incorporating advanced lipid profiling are required to clarify the clinical significance of these changes [25, 26].

FXR activation modulates cholesterol metabolism via several pathways. Studies show FXR agonism increases cholesteryl ester transfer protein, upregulates hepatic scavenger receptor class B type I and suppresses apolipoprotein A‐I transcription [22, 27, 28]. These changes may reduce HDL‐C and affect reverse cholesterol transport during therapy. However, whether these biochemical alterations ultimately translate into clinically meaningful increases in cardiovascular risk remains uncertain because long‐term cardiovascular outcome data for FXR agonists in MASLD remain limited.

Despite concerns regarding lipid alterations, OCA therapy produced significant improvements in hepatic biochemical parameters and noninvasive fibrosis‐related surrogate indices. These findings support the therapeutic potential of FXR activation in MASLD and are consistent with previous studies reporting histological and metabolic benefits of OCA in patients with MASH [13, 15]. Notably, the observed hepatic improvement occurred despite only modest reductions in systemic insulin resistance, suggesting that FXR agonism may exert direct hepatocellular metabolic and anti‐inflammatory effects independent of major peripheral insulin sensitisation. However, the simultaneous improvement in liver‐related parameters and elevation in LDL‐C highlights an important therapeutic paradox associated with FXR‐directed therapy. Although OCA improves steatosis and fibrosis‐related indices, it may also induce lipid alterations linked to ASCVD risk [18]. These findings emphasise the significance of regular lipid monitoring during FXR agonist therapy and suggest that concomitant lipid‐lowering therapy may be beneficial in selected patients.

The present study is strengthened by its relatively large sample size, balanced baseline characteristics, structured follow‐up and multivariable adjustment. Despite these strengths, several limitations should be acknowledged. First, MASLD diagnosis relied on abdominal ultrasonography, which has limited sensitivity for detecting mild steatosis and cannot accurately stage hepatic fibrosis. Because liver stiffness assessment using transient elastography, MRI‐PDFF, MRE or liver biopsy was not performed, direct assessment of hepatic fibrosis or fibrosis regression was not possible. Residual confounding resulting from unrecorded concomitant medications, including lipid‐lowering and antidiabetic therapies, cannot be excluded and may have influenced the observed metabolic outcomes. In addition, information regarding posttreatment statin initiation and the proportion of patients exceeding clinically relevant LDL‐C thresholds was unavailable, limiting further cardiovascular risk stratification.

## 5. Conclusion

In adults with MASLD, OCA combined with lifestyle modification produced significant short‐term improvements in liver enzymes and validated noninvasive fibrosis‐related surrogate indices compared with lifestyle modification alone. Because fibrosis was not directly assessed using elastography or liver biopsy, no conclusions regarding true fibrosis regression can be drawn. However, treatment was also associated with increased LDL cholesterol levels, highlighting the complex metabolic effects of FXR agonism. Although the hepatic benefits are promising, careful lipid surveillance and consideration of cardiovascular risk assessment remain essential during therapy. Larger randomised controlled trials with longer follow‐up incorporating transient elastography, MRI‐based imaging or histological assessment are required before definitive conclusions regarding fibrosis improvement and long‐term cardiovascular safety can be drawn.

## Author Contributions

All authors have critically reviewed the final draft.

## Funding

No funding was received for this manuscript.

## Disclosure

All authors approved the final draft and are responsible for the content and similarity index of the manuscript.

## Ethics Statement

The study was conducted in accordance with the Declaration of Helsinki and was approved by an ethics review committee (09‐08‐2024) (Reference No. 210‐Synopsis/III/FJ/ERC).

## Consent

Informed consent was taken from all patients who participated in this study.

## Conflicts of Interest

The authors declare no conflicts of interest.

## Data Availability

The data that support the findings of this study are available from the corresponding author upon reasonable request.
